# Bidirectional longitudinal relationships between MRI-detected structural changes and contralateral knee pain in knee osteoarthritis: Data from the Osteoarthritis Initiative

**DOI:** 10.1016/j.ocarto.2026.100778

**Published:** 2026-03-13

**Authors:** Shao-Hsien Liu, Jonggyu Baek, Kate L. Lapane, Julieann C. Patarini, Ming Zhang, Matthew S. Harkey, Grace H. Lo, Charles B. Eaton, Jamie MacKay, Timothy E. McAlindon, Jeffrey B. Driban

**Affiliations:** a Division of Epidemiology, Department of Population and Quantitative Health Sciences, UMass Chan Medical School, Worcester, MA 01655, USA; b Division of Biostatistics and Health Services Research, Department of Population and Quantitative Health Sciences, UMass Chan Medical School, Worcester, MA 01655, USA; c Division of Rheumatology, Department of Medicine, UMass Chan Medical School, Worcester, MA 01655, USA; d Department of Computer Science, Boston University, 111 Cummington Mall, Boston, MA 02215, USA; e Department of Kinesiology, Michigan State University, 308 W. Circle Drive, East Lansing, MI 48824, USA; f Department of Medicine, Baylor College of Medicine, One Baylor Plaza, Houston, TX 77030, USA; g Medical Care Line and Research Care Line, Houston VA HSR&D Center for Innovations in Quality, Effectiveness and Safety, Michael E. DeBakey Medical Center, 2002 Holcombe Blvd, Houston, TX 77030, USA; h Center for Primary Care and Prevention, Kent Hospital, 111 Brewster St, Pawtucket, RI 02860, USA; i Brown University School of Public Health, 121 South Main Street Providence, RI 02903, USA; j Department of Radiology, University of Cambridge, Box 218, Cambridge Biomedical Campus, Cambridge CB2 0QQ, United Kingdom; k Norwich Medical School, University of East Anglia, Norwich Research Park, Norwich NR4 7TJ, United Kingdom

**Keywords:** Disease activity, Magnetic resonance imaging, Bilateral knee, Knee osteoarthritis, Longitudinal study

## Abstract

**Objective:**

To examine bidirectional longitudinal associations between MRI-detected structural changes and pain between knees (within a person) with or at risk for osteoarthritis (OA).

**Design:**

Using data from the Osteoarthritis Initiative (OAI) with a bidirectional knee-based design, each participant contributed two observations by alternating one knee as the exposure knee and the other as the contralateral (outcome) knee. MRI-based composite metrics of disease activity and cumulative damage were assessed over two years. Knee pain was measured using The Western Ontario and McMaster Universities Osteoarthritis Index (WOMAC). Multinomial logistic regression estimated associations between baseline or changes in MRI from one knee and changes in the contralateral knee pain. Linear regression assessed associations between baseline or changes in structural metrics of one knee and changes in the contralateral knee.

**Results:**

The sample included 625 knee pairs (1250 knees) from 303 participants, with some contributing data from multiple intervals. Baseline measures or longitudinal changes in disease activity or cumulative damage in the one knee were not associated with two-year changes in pain in the contralateral knee. Two-year changes in disease activity (adjusted β = 0.16, 95% CI: 0.05 to 0.27) and cumulative damage (adjusted β = 0.16, 95% CI: 0.03 to 0.30) were associated with contralateral knee disease activity and cumulative damage changes, respectively.

**Conclusions:**

Structural worsening in one knee was not associated with contralateral pain but correlated with concurrent structural worsening in the other knee, suggesting parallel bilateral progression and the need to consider both knees in OA studies and therapeutic trials.

## 1 Introduction

Knee osteoarthritis (OA) is a leading cause of pain and disability, affecting >360 million people worldwide and imposing a substantial economic burden with annual costs exceeding $12 billion for total knee arthroplasty procedures alone [1,2]. Effective nonsurgical treatments remain limited, with current therapies offering only small-to-moderate effects and significant adverse events [3,4]. This therapeutic gap has led to concerning trends, including chronic opioid use among one in 10 Medicare beneficiaries with knee OA [5].

In knee OA research, the predominant focus is on unilateral disease, despite evidence that most individuals with symptomatic knee OA have bilateral involvement [6,7]. This singular knee approach may explain suboptimal treatment outcomes, as demonstrated in recent clinical trials where intra-articular lorecivivint improved outcomes in people with unilateral but not bilateral knee OA [8,9]. Our recent cross-sectional analysis of bilateral MRI data revealed that contralateral knee OA severity was associated with structural damage in the other knee, with bone marrow lesions showing particularly strong cross-knee effects [7]. Other investigators have demonstrated that radiographically normal knees with contralateral joint space narrowing experience greater articular cartilage changes than people with bilateral radiographically normal knees [[10], [11], [12]]. However, the complex bidirectional and longitudinal relationship between structural damage and symptoms changes between both knees remains poorly understood, potentially limiting therapeutic efficacy for the approximately 50% of patients with bilateral disease [[12], [13], [14], [15]].

Using data from the Osteoarthritis Initiative (OAI), a comprehensive longitudinal dataset with bilateral quantitative structural assessments [16], this study aimed to characterize longitudinal changes in knee pain and structure in both knees, examining their reciprocal influences between knees (within person) over time. We expanded on the existing literature by concurrently studying changes in knee pain and magnetic resonance imaging (MRI)-based structural changes, including bone marrow lesions (BMLs), effusion-synovitis, and articular cartilage damage. We hypothesized that structural damage in one knee would predict subsequent and concurrent worsening in the contralateral knee, and that this relationship would be bidirectional, reflecting a dynamic interplay between knees over time. By further exploring these bidirectional and longitudinal relationships, we seek to inform future therapeutic and OA monitoring strategies that address both knees simultaneously, ultimately improving outcomes for individuals with bilateral knee OA.

## 2 Method

This study was reviewed by the UMass Chan Medical School Institutional Review Board and considered exempt from human subjects research oversight.

### 2.1 Data source

We used data from OAI, a publicly available, multicenter, prospective cohort study designed to identify biomarkers and risk factors associated with the development and progression of knee OA. Between 2004 and 2006, the OAI staff enrolled 4796 participants aged 45–79 years with or at increased risk of symptomatic knee OA at four U.S. clinical sites. The study protocol included annual clinical assessments, imaging (MRI and radiographs), and patient-reported outcomes.

### 2.2 Study design and sample

We conducted longitudinal, knee-based analyses from a nested cohort of the OAI. Each participant contributed two observations with the bidirectional design by switching the exposure/outcome relationship of their knees. For instance, the right knee served as the exposure knee and the left as the contralateral knee (outcome) in one observation. In the second, the left knee served as the exposure knee and the right as the outcome knee. Our primary analyses did not reverse the roles of pain and MRI measures (i.e., we did not model pain as the exposure and MRI outcomes), but instead evaluated MRI measures in one knee in relation to pain or MRI measures in the contralateral knee.

To have the high-quality structural and symptomatic data on both knees for evaluating inter-knee relationships longitudinally, we included participants who met the following inclusion criteria: 1) had at least one knee with Kellgren-Lawrence (KL) grade ≥1 and WOMAC pain score ≥10/100; 2) no previous history of knee replacement through the 48-month visit; 3) had data available to assess changes in pain between the 12- and 48-month visits; 4) had bilateral knee MRIs at 3 or 4 consecutive visits; 5) had valid disease activity and cumulative damage metrics in both knees at the 12-month visit [7]. A total of 642 participants (1300 knees) were initially eligible. After excluding participants who lacked complete MRI or pain data across the required visits, the final analytic sample included 625 knee pairs (1250 knees) from 303 participants. Some knee pairs contributed more than one interval when eligible across consecutive visits defined by paired MRI and WOMAC pain assessments.

### 2.3 Magnetic resonance imaging

Bilateral knee MRIs were acquired at each OAI clinical site using Siemens 3.0 T Trio scanners with dedicated knee coils and a standardized imaging protocol. Quality control procedures were performed regularly by study-certified MRI technologists to ensure consistent image quality across sites and over time. Bone marrow lesion and effusion-synovitis volumes were quantified from intermediate-weighted fat-suppressed (IWFS) sequences (field of view 160 mm, slice thickness 3 mm, no gap, flip angle 180°, echo time 30 ms, repetition time 3200 ms, matrix 313 × 448, in-plane resolution 0.357 × 0.511 mm). Cartilage damage was quantified using a 3-dimensional dual-echo steady-state (3D DESS) sequence (field of view 140 mm, slice thickness 0.7 mm, skip 0 mm, flip angle 25°, echo time 4.7 ms, recovery time 16.3 ms, 307 × 384 matrix, in-plane resolution 0.365 × 0.456 mm) [17,18].

### 2.4 Measuring components of the disease activity and cumulative damage metrics

MRI-based disease activity was quantified using our validated composite metric, which integrates BML and effusion-synovitis volumes, both of which are dynamic features of knee OA strongly linked to pain and progression. BML volumes were measured in six anatomic regions, including medial and lateral compartments of the distal femur, proximal tibia, and patella, using semi-automated segmentation software with manual quality control on IWFS sequences [19]. Effusion-synovitis volume was measured on the same sequence, with thresholds manually adjusted to exclude non-relevant hyperintense areas (e.g., cysts, vessels) [20]. The primary effusion-synovitis volume represented effusion-synovitis throughout the entire knee. ntra-reader reliability for these measurements has been reported previously from an independent reliability study of this measurement protocol (intra-class correlation coefficients [3,1 model] >0.95; data not shown) [19,20].

We used a validated semi-automated program to determine the cartilage damage index (CDI), a parsimonious method to quantify tibiofemoral and patellar cartilage thickness at predetermined informative locations [21,22]. Using the DESS sequences, cartilage thickness was measured at 36 predetermined informative locations in the medial and lateral femur and tibia (i.e., 9 locations in each region), which the program automatically identified. Cartilage thickness was also measured at 24 predetermined informative locations in the medial and lateral patella (i.e., 12 locations in each region), which the program automatically identified [23]. The semi-automated program generated a CDI value for all six regions (medial and lateral femur, tibia, and patella). Reliability for CDI measurements has been evaluated previously using an independent reread reliability sample (intraclass correlation coefficient [3,1 model] = 0.84 to 0.98; data not shown) [[21], [22], [23]].

### 2.5 Calculating the disease activity and cumulative damage metrics

To calculate our metrics, all MRI-based volumes were adjusted for knee size using femoral bone width and then standardized using reference distributions derived from a predefined sample of OAI knees with and without OA. Disease activity was calculated using our validated algorithm and guided by stakeholder feedback [16]. The composite disease activity score was calculated as the sum of standardized BML and effusion-synovitis volumes. It is not bounded by a fixed theoretical minimum or maximum, with higher scores indicating greater active joint pathology.

MRI-based cumulative damage was assessed using the CDI, a validated quantitative measure of cartilage morphometry. CDI was calculated for the medial and lateral femur, tibia, and patella based on cartilage area and thickness extracted from DESS sequences. All CDI measures were adjusted for bone width, standardized using reference distributions, and standardized regional values were summed and multiplied by −1 to create a whole-knee cumulative damage score, with higher values representing greater structural damage.

### 2.6 Assessment of knee pain

We assessed knee pain using the knee-specific pain subscale of the Western Ontario and McMaster Universities Osteoarthritis Index (WOMAC, Likert version 3.1) [24]. Each of the five items in the pain subscale was scored from 0 (none) to 4 (extreme). Responses to each item were then summed to generate a pain score, yielding a range from 0 to 20. We then converted the original scale of the WOMAC pain score to 0 to 100. Higher scores indicate more severe pain. The primary outcome was the change in WOMAC pain scores between visits and was categorized into three groups: clinically meaningful improvement (<−20), no meaningful change (−20 to 20), and clinically meaningful worsening (>20) [25].

### 2.7 Covariates

Sociodemographics, including age and gender, were recorded at enrollment. Radiographic severity was assessed using Kellgren-Lawrence grade (KL). Trained central readers assigned KL grades (0–4) based on standardized, fixed-flexion posterior-anterior weight-bearing knee radiographs. Radiographic OA was defined as KL grade ≥2. Study staff measured each participant's height and weight. Body mass index was calculated as weight (kilograms) divided by height (meters squared) and classified as normal weight (18.5–24.9 kg/m^2^), overweight (25.0–29.9 kg/m^2^), or obese (≥30 kg/m^2^) [26]. The dominant side was determined based on self-reported preferred leg for activities such as kicking a ball.

### 2.8 Statistical analyses

Descriptive statistics were calculated for baseline characteristics, presented as percentages for categorical variables and means with standard deviations (SD) for continuous variables, stratified by categories of 2-year change in WOMAC pain in the contralateral knee. Each participant contributed two observations by reversing the exposure–outcome relationship between knees (e.g., right knee as exposure/left knee as contralateral (outcome) knee, and vice versa), and each knee contributed at least one follow-up interval to the analyses. For the purposes of this analysis, ‘baseline’ refers to the index visit (start) of each eligible 2-year interval for a given knee pair, rather than the OAI enrollment visit.

To examine the bidirectional longitudinal relationships between knees, we fit regression models accounting for correlations between knees within a participant. Specifically, we tested two primary hypotheses: (1) baseline or changes in MRI-based structural measures in one knee would be associated with ***changes in contralateral knee pain***, and (2) baseline or structural metrics would be associated with ***changes in contralateral structural metrics***. Multinomial logistic regression models with generalized estimating equations (GEEs) were used to estimate crude and adjusted odds ratios (ORs) with 95% confidence intervals (CIs) for associations between baseline or 2-year changes in MRI-based measures (disease activity and cumulative damage metrics; determinants of interest) in one knee (exposure) and categories of change in WOMAC pain in the contralateral knee (outcome), after accounting for knees within the same participant. Because these composite MRI metrics are standardized continuous measures developed for use in regression modeling, linear regression models with GEEs were used to estimate average cross-knee associations, accounting for knees within the same participant. To assess the influence of dominant side, we repeated the primary models stratified by whether the knee for the determinant of the interest was the dominant or non-dominant limb for sensitivity analyses. All analyses were two-sided, with p-values <0.05 considered statistically significant. Statistical analyses were performed using SAS version 9.4 (SAS Institute, Cary, NC) and R version 4.3.

## 3 Results

Table 1 summarizes the baseline characteristics stratified by categories of change in contralateral knee WOMAC pain over two years. Among 1250 knees (n = 303), 154 knees had meaningful pain improvement (76% female, 62% White, 64% obese, 84% radiographic knee osteoarthritis), 118 knees had worsening (74% female, 69% White, 65% obese, 83% radiographic knee osteoarthritis), and 978 had no meaningful change (65% female, 79% White, 51% obese, 81% radiographic knee OA). Baseline mean disease activity ranged from 0.19 to 0.60 across pain-change categories, and baseline mean cumulative damage ranged from 2.12 to 2.98.

**Table 1:** Baseline characteristics of included knees by 2-year change in WOMAC pain (n = 1250 knees).

	2-year changes of WOMAC^a^			
	Improvement			Worsening
	< −20 (n = 154)	−20 to < 0 (n = 540)	0 to 20 (n = 438)	>20 (n = 118)
Age ≥65 years (%)	36.4	42.6	41.1	39.0
Female (%)	76.0	69.4	60.5	73.7
Race (%)				
White	61.7	78.3	75.6	68.6
Black	35.1	21.1	21.7	29.7
Others	3.3	0.6	2.7	1.7
Body mass index, kg/m^2^ (%)				
<25.0	9.1	16.9	17.4	9.3
25.0–29.9	27.3	31.7	32.2	25.4
≥30.0	63.6	51.5	50.5	65.3
Dominant side, right (%)	85.4	90.5	90.8	90.2
Right knee (%)	55.8	50.9	46.1	52.5
Kellgren-Lawrence grade (%)				
1	15.6	18.9	18.7	17.0
2	52.6	47.4	46.4	33.9
3	26.6	29.1	25.6	43.2
4	5.2	4.6	9.4	5.9
Disease activity at baseline, mean (SD)	0.19 (3.03)	0.60 (3.60)	0.36 (3.00)	0.26 (2.80)
Cumulative damage at baseline, mean (SD)	2.98 (3.35)	2.46 (3.16)	2.69 (3.54)	2.12 (4.51)

a Negative score in WOMAC indicates improved symptoms.

Baseline MRI structural findings were not associated with subsequent two-year changes in contralateral pain and contralateral structural measures (Supplemental Tables 1 and 2). The associations of two-year changes in MRI-detected structural findings and contralateral knee pain are displayed in Table 2. Over two years, mean changes in disease activity ranged from 0.45 to 0.67, and mean changes in cumulative damage ranged from 0.62 to 0.93 across groups. Greater changes in MRI-assessed disease activity in the one knee were not associated with two-year changes in contralateral knee WOMAC pain. For each one-unit increase in 2-year cumulative damage score, there was increased odds of worsening pain (0–20 and > 20 points), although the 95% confidence interval included unity [adjusted OR (0–20): 1.11; 95% CI: 0.96 to 1.28; adjusted OR (>20): 1.21; 95% CI: 0.98 to 1.50].

**Table 2:** Associations of between MRI-detected two year change scores (disease activity, cumulative damage) and 2-year change in WOMAC pain (n = 1250 knees).

Outcome variable: 2-year changes of WOMAC^b^	Determinant: Mean MRI-detected structural change (SD)	Crude Odds Ratio (95% Confidence interval (CI))	Adjusted Odds Ratio^a^ (95% CI)
**Disease activity and contralateral knee pain**			
< −20	0.46 (2.88)	Reference	Reference
−20 to < 0	0.59 (2.47)	1.02 (0.92–1.13)	1.02 (0.92–1.13)
0 to 20	0.45 (2.21)	1.00 (0.90–1.11)	1.00 (0.89–1.12)
>20	0.67 (1.97)	1.04 (0.91–1.18)	1.03 (0.91–1.18)
**Cumulative damage and contralateral knee pain**			
< −20	0.62 (1.24)	Reference	Reference
−20 to < 0	0.82 (1.01)	1.14 (1.00–1.30)	1.10 (0.98–1.24)
0 to 20	0.83 (1.37)	1.08 (0.98–1.33)	1.11 (0.96–1.28)
>20	0.93 (0.91)	1.24 (1.00–1.55)	1.21 (0.98–1.50)

a Multinomial models with generalized estimating equations using 2-year WOMAC pain change (four levels) as the outcome and continuous MRI-based 2-year change scores (disease activity and cumulative damage in separate models) as predictors, adjusted for age, sex, race/ethnicity, BMI, KL grade, and knees within the same participant. For example, the odds of worsening knee pain (>20 points) were not significantly different from those of pain improvement per 2-year increase in cumulative damage (adjusted OR = 1.21; 95% CI: 0.98–1.50).

b Negative score in WOMAC indicates improved symptoms.

Cross-knee associations of MRI measures are shown in Table 3. Changes in disease activity in the one knee were associated with concurrent changes in contralateral disease activity (adjusted β = 0.16, 95% CI: 0.05 to 0.27). Similarly, changes in cumulative damage were associated with contralateral cumulative damage (adjusted β = 0.16, 95% CI: 0.03 to 0.30). No statistically significant cross-associations were observed between disease activity and cumulative damage across knees.

**Table 3:** Association of two-year bilateral MRI-detected structural changes (n = 1250 knees).

	Crude β (95% Confidence Interval)	Adjusted β^a^ (95% Confidence Interval)
**Outcome: 2-year change in contralateral disease activity**		
2-year change disease activity	0.16 (0.05–0.28)	0.16 (0.05–0.27)
2-year change cumulative damage	0.06 (−0.09 to 0.21)	0.05 (−0.09 to 0.19)
**Outcome: 2-year change in contralateral cumulative damage**		
2-year change disease activity	0.02 (−0.03 to 0.06)	0.01 (−0.03 to 0.05)
2-year change cumulative damage	0.17 (0.04–0.31)	0.16 (0.03–0.30)

a Derived from linear models with generalized estimating equations models using continuous MRI-based 2-year change scores (disease activity and cumulative damage in separate models) as predictors and 2-year changes in structural findings as outcomes, adjusted for age, gender, race/ethnicity, body mass index at baseline, and knees within the same participant.

### 3.1 Sensitivity analyses

When stratified by dominant side (Supplemental Tables 3 and 4), changes in disease activity of the dominant knee were associated with contralateral pain (adjusted β = 0.78, 95% CI: 0.001 to 1.57), while changes in cumulative damage of the non-dominant knee were associated with contralateral pain (adjusted β = 1.62, 95% CI: 0.45 to 2.79). Cross-knee associations of MRI measures were consistent across dominant and non-dominant knees (Supplemental Table 4). Changes in the disease activity metric in the one knee were associated with contralateral disease activity metric (dominant: adjusted β = 0.20, 95% CI: 0.06 to 0.33; non-dominant: adjusted β = 0.15, 95% CI: 0.05 to 0.26). Similarly, changes in the cumulative damage metric were associated with the contralateral cumulative damage metric (dominant: adjusted β = 0.15, 95% CI: −0.01 to 0.31; non-dominant: adjusted β = 0.17, 95% CI: 0.04 to 0.29).

## 4 Discussion

Using data from the OAI, we did not observe statistically significant associations between MRI-assessed disease activity or cumulative damage in one knee (baseline or changes) and changes in contralateral knee pain over two years, after adjusting for demographic and clinical covariates. In contrast, changes in disease activity and cumulative damage in one knee were significantly associated with corresponding changes in the contralateral knee, suggesting bilateral structural progression occurs in parallel. These findings were robust in sensitivity analyses, with results remaining generally consistent when stratified by dominant and non-dominant knees.

In this longitudinal study, we did not observe statically significant associations between baseline measures and changes in MRI-based disease activity or cumulative damage in one knee and changes in contralateral knee pain. These findings suggest that although systemic or biomechanical factors may affect both knees, structural worsening in one knee may not directly translate into pain worsening in the other. This contrasts with prior cross-sectional studies that reported associations between contralateral radiographic severity or bone marrow lesions and pain [7,27,28]. The lack of longitudinal associations may be due to factors such as the short follow-up period, temporal variability in pain reporting, or differential behavior of reversible inflammatory MRI features versus irreversible cartilage loss. In addition, pain may be a downstream consequence of structural worsening occurring first in the contralateral knee, such that a lagged relationship (i.e., structural change in one knee preceding contralateral structural change, which then precedes contralateral pain worsening) could extend beyond the same 2-year assessment window used here. Therefore, our concurrent 2-year change models may not capture delayed symptom responses to structural progression. Nevertheless, evidence from epidemiologic cohorts indicates that bilateral disease progression is common and asymmetric loading patterns are implicated in this progression [29,30]. Understanding these cross-knee interactions remains critical for developing treatment strategies and designing clinical trials, particularly for regenerative interventions that often focus on a single joint while many patients experience bilateral disease.

We observed that changes in MRI-detected disease activity and cumulative damage were significantly correlated between knees, supporting the concept of synchronous bilateral progression in knee OA. This pattern suggests that systemic drivers, such as low-grade inflammation or metabolic dysregulation, may underlie OA progression across both joints, rather than pathology being solely driven by localized joint-level factors [31,32]. Our findings are consistent with prior studies demonstrating bilateral cartilage loss, contralateral radiographic progression, and cross-knee associations of bone marrow lesions, which collectively point to shared biological mechanisms contributing to joint deterioration [7,30,33]. Recognition of bilateral structural progression underscores the need for therapeutic interventions targeting systemic contributors to OA and for clinical trial designs that account for bilateral involvement, since focusing on a single knee may not fully capture treatment effects.

Results from sensitivity analyses showed that disease activity of the dominant knee was associated with contralateral pain, whereas cumulative damage of the non-dominant knee showed associations with contralateral pain. These patterns suggest that loading characteristics and functional demands may differentially shape cross-knee relationships depending on whether the affected limb is dominant or non-dominant [[34], [35], [36]]. For example, the dominant knee may be more susceptible to activity-related inflammatory changes, while structural loss in the non-dominant knee may alter gait or weight distribution in ways that influence symptoms in the opposite joint. These findings highlight the potential importance of limb dominance as a modifier of bilateral OA relationships. More research is needed to understand whether stratifying or adjusting for limb dominance can improve characterization of inter-knee associations in bilateral OA.

This study has several strengths. We used data from the OAI, a large, well-characterized cohort with standardized longitudinal MRI assessments and validated composite measures of disease activity and cumulative damage. The bilateral design allowed us to evaluate bidirectional associations between knees within the same individual, which reduces confounding by shared systemic and behavioral factors. We also leveraged sensitivity analyses, such as stratification by dominant side, to confirm the robustness of our findings. Nonetheless, several limitations are acknowledged. The observational design precludes causal inference, and residual confounding by unmeasured factors such as physical activity patterns or limb loading cannot be excluded [[37], [38], [39]]. MRI-derived measures, while validated, may not capture all aspects of structural pathology relevant to pain [[40], [41], [42]]. In addition, follow-up was limited to two years with complete bilateral MRI and WOMAC data across eligible visits, which may be insufficient to detect longer-term cross-knee effects and resulted in exclusion of a substantial proportion of initially eligible participants and thus limiting generalizability. Moreover, because WOMAC pain and MRI were evaluated over the same 2-year intervals, we could not formally test lagged (cross-lagged) pathways in which structural worsening in one knee precedes contralateral structural change and subsequent contralateral pain changes. Future work with longer follow-up and lagged modeling is needed. Finally, while pain may plausibly influence subsequent structural progression (e.g., via altered gait/loading or activity modification), it was not evaluated as an exposure predicting contralateral MRI changes since it was considered more a temporally variable and may be influenced by non-structural factors.

In summary, MRI-assessed structural features of one knee (baseline and changes) were not associated with contralateral knee pain over two years, but changes in the structural metrics were significantly associated with corresponding structural changes in the other knee, indicating parallel bilateral progression. These findings highlight the importance of considering both knees when evaluating OA progression and suggest that future therapeutic strategies and clinical trial designs should account for bilateral disease processes.

## Author contributions

Study concept and design (Shao-Hsien Liu, Jeffrey Driban, Kate Lapane, Timothy McAlindon), data analysis (Jonggyu Baek, Shao-Hsien Liu), manuscript preparation (Shao-Hsien Liu, Jeffrey Driban), and interpretation of findings (all authors). All authors contributed to the interpretation of the data, preparation of the final manuscript, and revising it critically for important scientific content. The final version of this manuscript was approved by all authors.

## Declaration of Generative AI and AI-assisted technologies in the writing process

During the preparation of this work the authors used ChatGPT/OpenAI in order to improve the clarity and language of the manuscript. After using this tool/service, the authors reviewed and edited the content as needed and take full responsibility for the content of the publication.

## Role of funding source

This study was funded by the National Institute of Arthritis and Musculoskeletal and Skin Disease (Grant numbers: R01AR076411 and R24AR085006 awarded to Dr. Jeffrey Driban). The OAI is a public-private partnership comprised of five contracts (N01-AR-2-2258; N01-AR-2-2259; N01-AR-2-2260; N01-AR-2-2261; N01-AR-2-2262) funded by the National Institutes of Health, a branch of the Department of Health and Human Services, and conducted by the OAI Study Investigators. Private funding partners include Merck Research Laboratories; Novartis Pharmaceuticals Corporation, GlaxoSmithKline; and Pfizer, Inc. The funders had no role in design, methods, data collection, analysis, or preparation of this manuscript.

## Conflicts of interest

Jeffrey Driban reports financial support was provided by National Institute of Arthritis and Musculoskeletal and Skin Diseases. Jeffrey Driban reports a relationship with Sanofi that includes: consulting or advisory. Timothy McAlindon reports a relationship with Sanofi that includes: consulting or advisory. Timothy McAlindon reports a relationship with Kolon TissueGene that includes: consulting or advisory. Timothy McAlindon reports a relationship with Medidata that includes: consulting or advisory. Timothy McAlindon reports a relationship with Organogenesis Inc that includes: consulting or advisory. Timothy McAlindon reports a relationship with Ambulomics that includes: equity or stocks. Timothy McAlindon reports a relationship with Arthrometrics that includes: equity or stocks. Jeffrey Driban and Timothy McAlindon have a patent, Objective Assessment of Joint Damage (US-20220202356). All other authors declare that they have no known competing financial interests or personal relationships that could have appeared to influence the work reported in this paper.

## References

[1] Yang G., Wang J., Liu Y. (2023). Burden of knee osteoarthritis in 204 countries and territories, 1990–2019: results from the global burden of disease study 2019. Arthritis Care Res..

[2] Losina E., Paltiel A.D., Weinstein A.M. (2015). Lifetime medical costs of knee osteoarthritis management in the United States: impact of extending indications for total knee arthroplasty. Arthritis Care Res..

[3] GBD 2019 Diseases and Injuries Collaborators (2020). Global burden of 369 diseases and injuries in 204 countries and territories, 1990-2019: a systematic analysis for the global burden of disease study 2019. Lancet.

[4] da Costa B.R., Pereira T.V., Saadat P. (2021). Effectiveness and safety of non-steroidal anti-inflammatory drugs and opioid treatment for knee and hip osteoarthritis: network meta-analysis. BMJ.

[5] Losina E., Song S., Bensen G.P., Katz J.N. (2023). Opioid use among medicare beneficiaries with knee osteoarthritis: prevalence and correlates of chronic use. Arthritis Care Res..

[6] Riddle D.L., Stratford P.W. (2013). Unilateral vs bilateral symptomatic knee osteoarthritis: associations between pain intensity and function. Rheumatology.

[7] Driban J.B., Baek J., Patarini J.C. (2025). Contralateral knee osteoarthritis severity relates to magnetic resonance imaging findings in knees with and without osteoarthritis: data from the osteoarthritis initiative. Osteoarthr Cartil Open.

[8] Yazici Y., McAlindon T.E., Gibofsky A. (2021). A phase 2b randomized trial of lorecivivint, a novel intra-articular CLK2/DYRK1A inhibitor and wnt pathway modulator for knee osteoarthritis. Osteoarthr. Cartil..

[9] Yazici Y., McAlindon T.E., Gibofsky A. (2020). Lorecivivint, a novel intraarticular CDC-like kinase 2 and dual-specificity tyrosine phosphorylation-regulated kinase 1A inhibitor and Wnt Pathway modulator for the treatment of knee osteoarthritis: a phase II randomized trial. Arthritis Rheumatol..

[10] Eckstein F., Maschek S., Roemer F.W., Duda G.N., Sharma L., Wirth W. (2019). Cartilage loss in radiographically normal knees depends on radiographic status of the contralateral knee - data from the Osteoarthritis initiative. Osteoarthr. Cartil..

[11] Wirth W., Maschek S., Roemer F.W., Sharma L., Duda G.N., Eckstein F. (2019). Radiographically normal knees with contralateral joint space narrowing display greater change in cartilage transverse relaxation time than those with normal contralateral knees: a model of early OA? - data from the osteoarthritis initiative (OAI). Osteoarthr. Cartil..

[12] Cotofana S., Benichou O., Hitzl W., Wirth W., Eckstein F. (2014). Is loss in femorotibial cartilage thickness related to severity of contra-lateral radiographic knee osteoarthritis?--longitudinal data from the osteoarthritis initiative. Osteoarthr. Cartil..

[13] Lethbridge-Cejku M., Tobin J.D., Scott W.W., Reichle R., Plato C.C., Hochberg M.C. (1994). The relationship of age and gender to prevalence and pattern of radiographic changes of osteoarthritis of the knee: data from Caucasian participants in the Baltimore longitudinal study of aging. Aging (Milano).

[14] Cotofana S., Wirth W., Pena Rossi C., Eckstein F., Günther O.H. (2015). Contralateral knee effect on self-reported knee-specific function and global functional assessment: data from the osteoarthritis initiative. Arthritis Care Res..

[15] Steidle-Kloc E., Wirth W., Glass N.A. (2015). Is pain in one knee associated with isometric muscle strength in the contralateral limb?: data from the osteoarthritis initiative. Am. J. Phys. Med. Rehabil..

[16] Driban J.B., Price L.L., LaValley M.P. (2022). Novel framework for measuring whole knee osteoarthritis progression using magnetic resonance imaging. Arthritis Care Res..

[17] Frobell R.B. (2011). Change in cartilage thickness, posttraumatic bone marrow lesions, and joint fluid volumes after acute ACL disruption: a two-year prospective MRI study of sixty-one subjects. J Bone Joint Surg Am.

[18] Frobell R.B., Roos H.P., Roos E.M. (2008). The acutely ACL injured knee assessed by MRI: are large volume traumatic bone marrow lesions a sign of severe compression injury?. Osteoarthr. Cartil..

[19] Zhang M., Driban J.B., Price L.L., Lo G.H., McAlindon T.E. (2015). Magnetic resonance image sequence influences the relationship between bone marrow lesions volume and pain: data from the osteoarthritis initiative. BioMed Res. Int..

[20] Davis J.E., Ward R.J., MacKay J.W. (2019). Effusion-synovitis and infrapatellar fat pad signal intensity alteration differentiate accelerated knee osteoarthritis. Rheumatology.

[21] Zhang M., Driban J.B., Price L.L., Lo G.H., Miller E., McAlindon T.E. (2015). Development of a rapid cartilage damage quantification method for the lateral tibiofemoral compartment using magnetic resonance images: data from the osteoarthritis initiative. BioMed Res. Int..

[22] Zhang M., Driban J.B., Price L.L. (2014). Development of a rapid knee cartilage damage quantification method using magnetic resonance images. BMC Muscoskelet. Disord..

[23] Zhang M., Canavatchel A.R., Driban J.B. (2016). Patellar cartilage change is not associated with difficulty with stairs and crepitus: a two year longitudinal study of data from the osteoarthritis initiative. Osteoarthr. Cartil..

[24] Roos E.M., Klässbo M., Lohmander L.S. (1999). WOMAC osteoarthritis index. Reliability, validity, and responsiveness in patients with arthroscopically assessed osteoarthritis. Western Ontario and MacMaster universities. Scand. J. Rheumatol..

[25] Conaghan P.G., Dworkin R.H., Schnitzer T.J. (2022). WOMAC meaningful within-patient change: results from 3 studies of tanezumab in patients with moderate-to-severe osteoarthritis of the hip or knee. J. Rheumatol..

[26] Jensen Michael D., Ryan Donna H., Apovian Caroline M. (2013). 2013 AHA/ACC/TOS guideline for the management of overweight and obesity in adults. Circulation.

[27] Aso K., Shahtaheri S.M., McWilliams D.F., Walsh D.A. (2021). Association of subchondral bone marrow lesion localization with weight-bearing pain in people with knee osteoarthritis: data from the osteoarthritis initiative. Arthritis Res. Ther..

[28] Dai Z., Yang T., Liu J. (2024). Contralateral knee osteoarthritis is a risk factor for ipsilateral knee osteoarthritis progressing: a case control study. BMC Muscoskelet. Disord..

[29] Metcalfe A.J., Andersson M.L.E., Goodfellow R., Thorstensson C.A. (2012). Is knee osteoarthritis a symmetrical disease? Analysis of a 12 year prospective cohort study. BMC Muscoskelet. Disord..

[30] Wu R., Fu G., Li M. (2022). Contralateral advanced radiographic knee osteoarthritis predicts radiographic progression and future arthroplasty in ipsilateral knee with early-stage osteoarthritis. Clin. Rheumatol..

[31] Knights A.J., Redding S.J., Maerz T. (2023). Inflammation in osteoarthritis: the latest progress and ongoing challenges. Curr. Opin. Rheumatol..

[32] Robinson W.H., Lepus C.M., Wang Q. (2016). Low-grade inflammation as a key mediator of the pathogenesis of osteoarthritis. Nat. Rev. Rheumatol..

[33] Wluka A.E., Hanna F., Davies-Tuck M. (2009). Bone marrow lesions predict increase in knee cartilage defects and loss of cartilage volume in middle-aged women without knee pain over 2 years. Ann. Rheum. Dis..

[34] Nakahira Y., Taketomi S., Kawaguchi K. (2022). Kinematic differences between the dominant and nondominant legs during a single-leg drop vertical jump in female soccer players. Am. J. Sports Med..

[35] Zumstein F., Centner C., Ritzmann R. (2022). How limb dominance influences limb symmetry in ACL patients: effects on functional performance. BMC Sports Sci. Med. Rehabil..

[36] Mercado-Palomino E., Aragón-Royón F., Richards J., Benítez J.M., Ureña Espa A. (2021). The influence of limb role, direction of movement and limb dominance on movement strategies during block jump-landings in volleyball. Sci. Rep..

[37] Yang L., Wang P., McGill B. (2023). The relationship between experience of knee pain and physical activity participation: a scoping review of quantitative studies. Int. J. Nurs. Sci..

[38] Lazaridou A., Martel M.O., Cornelius M. (2019). The association between daily physical activity and pain among patients with knee osteoarthritis: the moderating role of pain catastrophizing. Pain Med..

[39] Wan Y., McGuigan P., Bilzon J., Wade L. (2025). Knee loading and joint pain during daily activities in people with knee osteoarthritis: a systematic review and meta-analysis. Clin. Biomech..

[40] Magnusson K., Turkiewicz A., Kumm J., Zhang F., Englund M. (2021). Relationship between magnetic resonance imaging features and knee pain over six years in knees without radiographic osteoarthritis at baseline. Arthritis Care Res..

[41] Smith S.E., Lo L., Sury M. (2025). Analysis of multiple MRI-Based quantitative structural measurements of knee osteoarthritis in a case control study - association with pain and structural progression and comparison to semi-quantitative scoring. Osteoarthr Cartil Open.

[42] Zhao Z., Zhao M., Yang T. (2024). Identifying significant structural factors associated with knee pain severity in patients with osteoarthritis using machine learning. Sci. Rep..

