# Supplementary material for: Bidirectional longitudinal relationships between MRI-detected structural changes and contralateral knee pain in knee osteoarthritis: Data from the Osteoarthritis Initiative
Source: Osteoarthr Cartil Open. 2026 Mar 13;8(2):100778. doi: 10.1016/j.ocarto.2026.100778 (PMC13058964; doi:10.1016/j.ocarto.2026.100778)
Supplement: Multimedia component 1 [file mmc1.docx]

Liu SH, Baek J, Lapane KL, Patarini JC, Zhang M, Harkey MS, Lo GH, Eaton CB, McAlindon TE, Driban JB. Bidirectional longitudinal relationships between MRI-detected structural changes and contralateral knee pain in knee osteoarthritis: data from the Osteoarthritis Initiative.

| **Topic** | **Item** | **Page** |
| --- | --- | --- |
| Sensitivity analysis | **Supplemental Table 1.** Association between baseline MRI-detected structural findings and 2-year changes in contralateral knee pain (n=1,250 knees). | 2 |
| Sensitivity analysis | **Supplemental Table 2.** Association between baseline and 2-year changes in MRI-detected structural findings (n=1,250 knees). | 3 |
| Sensitivity analysis | **Supplemental Table 3.** Association of 2-year change in MRI-detected structural findings and 2-year change in contralateral knee pain stratified by study knee dominant side (n=1,250 knees). | 4 |
| Sensitivity analysis | **Supplemental Table 4.** Association of 2-year change in bilateral MRI-detected structural changes stratified by study knee dominant side(n=1,250 knees). | 5 |

**Supplemental Table 1.** Association between baseline MRI-detected structural findings and 2-year changes in contralateral knee pain (n=1,250 knees).

| Outcome variable:  2-year changes of WOMAC± | Crude Odds Ratio  (95% Confidence Interval) | Adjusted Odds Ratio*  (95% Confidence Interval) |
| --- | --- | --- |
|  |  |  |
| **Disease activity at baseline and contralateral knee pain** | | |
| < -20 | Ref | Ref |
| -20 to < 0 | 1.04  (0.96 to 1.13) | 1.05  (0.96 to 1.16) |
| 0 to 20 | 1.02  (0.93 to 1.11) | 1.03  (0.93 to 1.13) |
| >20 | 1.01  (0.91 to 1.12) | 1.00  (0.89 to 1.13) |
| **Cumulative damage at baseline and contralateral knee pain** | | |
| < -20 | Ref | Ref |
| -20 to < 0 | 0.96  (0.90 to 1.02) | 0.98  (0.92 to 1.05) |
| 0 to 20 | 0.98  (0.91 to 1.05) | 1.03  (0.95 to 1.11) |
| >20 | 0.93  (0.83 to 1.05) | 0.92  (0.81 to 1.05) |

* Multinomial models with generalized estimating equations using 2-year WOMAC pain change (four levels) as the outcome and continuous MRI-based scores at baseline (disease activity and cumulative damage in separate models) as predictors, adjusted for age, sex, race/ethnicity, BMI, KL grade, and knees within the same participant.

± Negative score in WOMAC indicates improved symptoms.

**Supplemental Table 2.** Association between baseline and 2-year changes in MRI-detected structural findings (n=1,250 knees).

|  | Crude β  (95% Confidence Interval) | Adjusted β*  (95% Confidence Interval) |
| --- | --- | --- |
| **Outcome: 2-year change in contralateral disease activity** | | |
| Baseline disease activity | -0.01  (-0.11 to 0.08) | -0.01  (-0.10 to 0.09) |
| Baseline cumulative damage | 0.05  (-0.01 to 0.11) | 0.06  (-0.002 to 0.12) |
| **Outcome: 2-year change in contralateral cumulative damage** | | |
| Baseline disease activity | -0.002  (-0.03 to 0.03) | -0.002  (-0.03 to 0.02) |
| Baseline cumulative damage | -0.02  (-0.04 to 0.01) | -0.01  (-0.04 to 0.01) |

* Derived from linear models with generalized estimating equations models using continuous MRI-based scores at baseline (disease activity and cumulative damage in separate models) as predictors and 2-year changes in structural findings as outcomes, adjusted for age, gender, race/ethnicity, body mass index at baseline, and knees within the same participant.

**Supplemental Table 3.** Association of 2-year change in MRI-detected structural findings and 2-year change in contralateral knee pain stratified by study knee dominant side (n=1,250 knees).

| 2-year change in disease activity and 2-year change in contralateral knee pain± | | |
| --- | --- | --- |
|  | Crude β  (95% Confidence Interval) | Adjusted β*  (95% Confidence Interval) |
| Dominant¥ | 0.83  (0.04 to 1.62) | 0.78  (0.001 to 1.57) |
| Non-dominant | -0.16  (-0.77 to 0.45) | -0.15  (-0.75 to 0.45) |
| 2-year change in cumulative damage and 2-year change in contralateral knee pain± | | |
| Dominant | -0.06  (-1.19 to 1.07) | -0.14  (-1.24 to 0.97) |
| Non-dominant | 1.66  (0.50 to 2.82) | 1.62  (0.45 to 2.79) |

* Derived from linear models with generalized estimating equations models using continuous MRI-based 2-year change scores (disease activity and cumulative damage in separate models) as predictors and 2-year changes in WOMAC pain as outcomes, adjusted for age, gender, race/ethnicity, body mass index at baseline, and knees within the same participant.

± Negative score in WOMAC indicates improved symptoms

¥ n = 596 for both dominant and non-dominant measurements.

**Supplemental Table 4.** Association of 2-year change in bilateral MRI-detected structural changes stratified by study knee dominant side(n=1,250 knees).

|  | Crude β  (95% Confidence Interval) | Adjusted β*  (95% Confidence Interval) |
| --- | --- | --- |
| 2-year change in disease activity and 2-year change in contralateral disease activity | | |
| Dominant¥ | 0.19  (0.05 to 0.33) | 0.20  (0.06 to 0.33) |
| Non-dominant | 0.15  (0.05 to 0.26) | 0.15  (0.05 to 0.26) |
| 2-year change in cumulative damage and 2-year change in contralateral cumulative damage | | |
| Dominant | 0.16  (-0.02 to 0.33) | 0.15  (-0.01 to 0.31) |
| Non-dominant | 0.17  (0.05 to 0.29) | 0.17  (0.04 to 0.29) |

* Derived from linear models with generalized estimating equations models using continuous MRI-based 2-year change scores (disease activity and cumulative damage in separate models) as predictors and 2-year changes in structural findings as outcomes, adjusted for age, gender, race/ethnicity, body mass index at baseline, and knees within the same participant.

¥ n = 596 for both dominant and non-dominant measurements.
